# Down syndrome regression disorder, a case series: Clinical characterization and therapeutic approaches

**DOI:** 10.3389/fnins.2023.1126973

**Published:** 2023-02-23

**Authors:** Sidney Bonne, Anton Iftimovici, Clotilde Mircher, Martine Conte, Cécile Louveau, Adrien Legrand, Charlotte Danset-Alexandre, Costanza Cannarsa, Alexis Debril, Angèle Consoli, Marie-Odile Krebs, Pierre Ellul, Boris Chaumette

**Affiliations:** ^1^Centre de Référence pour les Maladies Rares à Expression Psychiatrique, GHU Paris Psychiatrie et Neurosciences, Paris, France; ^2^Université Paris Cité, Institute of Psychiatry and Neuroscience of Paris (IPNP), INSERM U1266, Paris, France; ^3^Institut Jérôme Lejeune, Paris, France; ^4^EDRPsy, UMR 5229, Centre National de la Recherche Scientifique (CNRS), Paris, France; ^5^Department of Child and Adolescent Psychiatry, Hospital Robert Debré Ap-Hp, Paris, France; ^6^Department of Child and Adolescent Psychiatry, Pitié-Salpêtrière Hospital, Paris, France; ^7^Dimensional Approach of Child and Adolescent Psychotic Episodes, Faculté de Médecine, Sorbonne Université, Paris, France; ^8^Immunology-Immunopathology-Immunotherapy (i3), UMRS 959, INSERM, Paris, France; ^9^Department of Psychiatry, McGill University, Montreal, QC, Canada

**Keywords:** down syndrome, trisomy 21, catatonia, inflammation, corticosteroids, personalized medicine

## Abstract

Down syndrome (DS) is one of the most frequent genetic disorders and represents the first cause of intellectual disability of genetic origin. While the majority of patients with DS follow a harmonious evolution, an unusual neurodevelopmental regression may occur, distinct from that described in the context of autism spectrum disorders, called down syndrome regression disorder (DSRD). Based on four patients, two males and two females, with age range between 20 and 24, treated at the Reference Center for Rare Psychiatric Disorders of the GHU Paris Psychiatry and Neurosciences [Pôle hospitalo-universitaire d’Évaluation Prévention et Innovation Thérapeutique (PEPIT)], we describe this syndrome, discuss its etiologies and propose therapeutic strategies. DSRD often occurs in late adolescence. There is a sudden onset of language disorders, loss of autonomy and daily living skills, as well as behavioral symptoms such as depression, psychosis, or catatonia. These symptoms are non-specific and lead to an overlap with other diagnostic categories, thus complicating diagnosis. The etiologies of the syndrome are not clearly identified but certain predispositions of patients with trisomy 21 have suggested an underlying immune-mediated mechanism. Symptomatic therapeutic approaches (serotonergic antidepressants, atypical antipsychotics, benzodiazepines) were not effective, and generally associated with poor tolerance. Etiological treatments, including anti-inflammatory drugs and corticosteroids, led to partial or good recovery in the four cases. Early recognition of regressive symptoms and rapid implementation of adapted treatments are required to improve the quality of life of patients and their families.

## Introduction

Down syndrome (DS), has an incidence of 1 per 800 births worldwide (Bull, 2020). In 1959, Lejeune, Gauthier, and Turpin described the association between DS and a third copy of chromosome 21, reporting for the first time in a disease a link between genotype and phenotype (Lejeune et al., 1959). Management of patients with DS significantly improved, with a constant increase in life expectancy, gaining more than 25 years (Antonarakis et al., 2020). Classical morphological features include facial dysmorphia, flattened occiput, microcephaly, single palmar crease, clinodactyly of the 5th finger, ligament and skin hyperlaxity, and overall muscle hypotonia. From a neurodevelopmental perspective, DST21 represents the leading cause of intellectual disability of genetic origin, and is often associated with autism spectrum and attention-deficit hyperactivity disorders, with high interindividual phenotypic variability and frequent co-occurrence of other psychiatric disorders (psychotic and mood disorders) (Antonarakis et al., 2020). Medical complications include cardio-pulmonary, hearing, oncologic, musculoskeletal, and notably, autoimmune disorders (Bull, 2020). In particular, thyroid-related disorders are estimated at 24% in DS children and 50% in adults (Pierce et al., 2017).

Although a majority of patients with DS follow a harmonious evolution, an adolescent behavioral regression may occur, making it distinct from those described earlier on in autism spectrum disorders. It occurs around 20 years old (18 years for girls and 21 years for boys), compared with around 5 years for DS patients with ASD and 20 months for autistic patients without DS (Worley et al., 2015). This entity, called down syndrome regression disorder (DSRD), is increasingly reported in the medical literature. There is a female predominance in DSRD, 64% being female as compared to the male predominance in ASD associated or not with DS (70–80%) (Castillo et al., 2008; Mircher et al., 2017). Regression occurs regardless of the level of intellectual disability and is not correlated with patients’ cognitive performance (Mircher et al., 2017). It is characterized by the acute onset, in adolescence, of a loss of previously acquired social and communicative skills, a loss of autonomy in activities of daily living (dressing, toileting, eating, continence), and disruption of executive functions (memory, attention, processing speed) and motor skills (appearance of abnormal movements, stereotypies, perseveration, extrapyramidal symptoms, catatonic syndrome) (Rosso et al., 2020).

While the exact prevalence of DSRD is unknown, more than 40 cases have been described in the literature (Miles et al., 2019; Santoro et al., 2020), leading to a better recognition of this condition that may not be especially rare. A recent international expert consensus established a nomenclature, clinical diagnostic criteria, and first-line complementary tests (Santoro et al., 2022b). DSRD is now used instead of Down Syndrome Disintegrative Disorder (DSDD) and Unexplained Regression in Down Syndrome (URDS). Diagnostic criteria were classified in 8 dimensions, of which three make the diagnosis possible, and six probable. They include: (1) an altered mental status or behavioral dysregulation, (2) a cognitive decline, (3) a developmental regression with or without new autistic features, (4) new focal neurologic deficits on examination or seizures, (5) insomnia or circadian rhythm disruption, (6) language deficits, (7) movement disorders (excluding tics), and (8) psychiatric symptoms.

Symptom onset needs to be sudden, over a period of less than 12 weeks in a previously healthy individual with DS, in the absence of alternative causes (Santoro et al., 2022b). Some symptoms are more frequently observed than others.

(i) Approximately 90% of patients with DSRD showed language regression, with symptoms ranging from dysfluency (38%) to mutism (52%) (Mircher et al., 2017). A change in voice (sometimes barely audible whispering) and a slowing of verbal fluency are often observed.
(ii) Psychiatric syndromes such as mood disorders are reported in 42% of cases (apathy, abulia, sadness of mood), followed by social withdrawal (34%) and anxiety disorders (16%). In addition, 14% of psychotic symptoms, including delusions, hallucinations, soliloquies, and unmotivated laughter are found in patients with DSRD. Behavioral disorders are frequent, with aggressive behavior occurring in 42% of patients (Mircher et al., 2017).
(iii) A marked psychomotor slowing down, sometimes as part of a catatonic syndrome, is often described. The clinical picture frequently includes sudden-onset abnormal movements such as stereotypies, tics and grimaces, or daytime bruxism. In the literature, a catatonic presentation is predominant in these patients at the time of diagnosis of DSRD. The catatonic syndrome, as a mode of revelation of DSRD, may concern 37% of patients (Mircher et al., 2017).
(iv) Sleep disorders are frequent, including inversions of the nycthemeral rhythm and insomnia. Sudden onset insomnia has been described in about 40% of cases of DSRD (Worley et al., 2015). This inaugural sleep disorder appears to be a characteristic feature of DSRD, often reported by parents. Finally, anorexia nervosa is a comorbidity affecting about 12% of patients with DSRD (Rosso et al., 2020).

Regarding the natural course of DSRD, an acute phase of six months is followed by a chronic phase of variable duration, after which previously acquired skills may not be fully recovered. Thus, 58% of people with DSRD experienced partial or total recovery, 7% of patients experienced further deterioration, while 35% of patients stabilized. However, in patients with stabilized DSRD, full recovery to the premorbid baseline appears to be uncommon (Rosso et al., 2020).

Based on these consensual diagnostic criteria, we reported here a series of four clinical situations of probable DSRD, and we described the therapeutic measures proposed. While the previously reported cases underwent invasive therapy, including electroconvulsive therapy or intravenous immunoglobulin, we propose to use less aggressive drugs, including corticosteroid or non-steroid anti-inflammatory drugs, with satisfying outcomes.

## Methods

Between December 2021 and December 2022, we included four patients with DS who presented criteria for DSRD and were seen at the reference center for rare diseases with psychiatric expression (CRMR, PEPIT department, GHU Paris Psychiatry and Neurosciences). Diagnosis of DSRD was done by two expert psychiatrists (PE and BC) according to the Expert Consensus (Santoro et al., 2022b) and after reasonable exclusion of alternative causes of regression, including primary psychiatric disorders. All patients were referred from the Jérôme Lejeune Institute in Paris, where patients were regularly followed. The Jérôme Lejeune institute is an expert center specialized in healthcare and research for individuals with DS. All patients and their families gave their consent for publication of this case series. Clinical presentations, the main workup, and the therapeutic challenges of the four cases are summarized in the Table 1 below.

**TABLE 1:** Description of the four cases: age, sex, clinical criteria and score for DSRD according to the expert consensus, paraclinical workup, treatments (+: presence; −: absence).

	Case 1	Case 2	Case 3	Case 4
Age	20	24	24	22
Sex	F	F	M	M
(1) Altered mental status or behavioral dysregulation	+ (disorientation, anorexia, inappropriate laughter)	+ (hyporexia, confusion)	+ (disorientation)	+ (disorientation)
(2) Cognitive decline	+ (apathy)	+ (avolition)	+ (acute memory impairment)	+ (apathy)
(3) Developmental regression with or without new autistic features	+ (loss of previously developmental acquired milestones such as drawing and writing)	+ (inability to perform activities of daily living, social withdrawal)	+ (loss of previously developmental acquired milestones such as enuresis, inability to perform activities of daily living, motor stereotypy)	+ (Loss of previously developmental acquired milestones such writing)
(4) New focal neurologic deficits on examination and/or seizure	–	–	–	–
(5) Insomnia or circadian rhythm disruption	+ (insomnia)	+ (insomnia, wake up during the night)	+ (irregular sleep cycles at polysomnography)	+ (insomnia)
(6) Language deficits	+ (partial mutism)	+ (whispered speech, verbigerations)	+ (mutism)	+ (major stammering)
(7) Movement disorder	+ (slowliness)	–	+(catatonia)	+ (catatonia)
(8) Psychiatric symptoms	+ (hallucinations)	+ (persecutive delusion)	+ (anxiety and depression)	+ (anxiety)
Total score	7 (probable DSRD)	6 (probable DSRD)	7 (probable DSRD)	6 (probable DSRD)
Duration before adequate treatment	9 months	22 months	4 years	6 years
Rapidity of onset	<24 h	2 weeks	4 weeks	4 weeks
MRI	Normal	Normal	Normal	Normal
EEG	Normal	Normal	Normal	Normal
Biological workup for inflammation/auto-immunity	Hypergammaglobulinemia No auto-antibodies (antinuclear, anti-thyroid) Normal level of C-reactive protein	Thyroperoxidase antibodies (>13,000 IU/ml) and anti-TRAK (30.6 UI/ml)	Anti-thyroglobulin antibodies (193 IU/ml)	Anti-thyroglobulin antibodies (9.6 IU/ml)
Anti-inflammatory treatment	Good response to naproxen 275 mg x 2/d	Good response to 3 days of IV corticoids followed by oral corticotherapy (prednisone 1 mg/kg/d) with progressive decrease during 8 months	Partial response to naproxen 550 mg x 2/d	No efficacy of naproxen 27 5 mg x 2/d Partial response to oral corticotherapy (prednisone 1 mg/kg/d)
Other treatments	Risperidone 1 mg/d had adverse reactions (worsening of psychomotor regression and lingual dyskinesias) and was stopped	Low doses of aripiprazole (1–2 mg/d) were efficient on screaming but increased apathy Previous challenge with valproic acid was unsuccessful.	ISRS (fluoxetine, sertraline) were initially efficient but then resistance occurs Lormetazepam 2 mg/d not efficient and not tolerated (sleepiness)	Lorazepam (1–2 mg/d) and ISRS (fluoxetine, sertraline) partially effective. Fluoxetine was associated with HyperStudio

## Case series

### Case 1

A 20-year-old woman with DS was referred to our tertiary center for a sudden onset of visual and auditory hallucinations. Her previous medical history was unremarkable. She had a yearly medical check-up for her DS. She lived with her mother and had an adapted schooling. At age 20, in less than 24 h; she presented severe behavioral symptoms: auditory hallucinations with soliloquy, fits of anger, unmotivated laughter, a fixed gaze, and insomnia. There was a psychomotor regression, with loss of graphic abilities, perseverations (doubling of letters at the end of words), tics, repetitive and abnormal eye and mouth movements, as well as partial mutism. During interviews, the patient’s speech was poor and undetailed, but she explained seeing dragons and having pain in her ears. Her mother also reported a loss of appetite with weight loss, a loss in sphincter control, a dramatic decrease in autonomy in terms of daily living skills (dressing) and praxis difficulties, particularly losing the ability to use a knife. In this context, the family consulted the emergencies several times, which referred them to the outpatient psychiatric consultation associated with their place of residence. A treatment with risperidone 2 mg/day was introduced. Despite an improvement in psychotic symptoms, it had to be reduced to risperidone 1 mg/day with the addition of tropatepine 5 mg, due to a worsening of the psychomotor regression and occurrence of lingual dyskinesias. It allowed a quick symptomatic improvement, and treatment cessation was proposed three months later. However, the patient soon after presented with an episode of diarrhea and vomiting with prostration, followed by the reappearance of behavioral problems. An electroencephalogram (EEG) showed a well-spatialized background pattern, with a few acute right temporo-occipital slow irregularities possibly related to artifacts and no epileptic abnormalities. The brain magnetic resonance imaging (MRI) was also normal. Upon referral to our center, the diagnostic hypothesis of DSRD related to Pediatric Acute-onset Neuropsychiatric Syndrome (PANS) was considered. A biological workup was ordered including: blood count, serum protein electrophoresis, thyroid hormonology, thyroid ANA and anti-soluble antibody assays. Only hypergammaglobulinemia was found at 16 g/L. In this context and in the hypothesis of a PANS, a treatment with a non-steroidal anti-inflammatory drug was started - naproxen 275 mg twice a day (10 mg/kg). This treatment improved the language problems and the psychomotor slowing down after 1 week, but the initial cognitive abilities have not yet been completely recovered. Naproxen cessation was followed each time by a relapse, leading to prolonged use. A total of 1 year after starting naproxen, the patient remains stable.

### Case 2

A 24-year-old woman with DS was referred to our center for anxiety attacks, incoherent speech, swallowing problems without hyporexia or general alteration. There were also attention problems, a loss of enthusiasm, and a marked psychomotor slowdown. While she was autonomous in daily life, she became dependent on assistance for washing and dressing. Episodes of encopresis occurred. Her previous difficulties in speech and language increased, making it difficult for her to communicate verbally, and isolating her from conversations with friends. She was very affected by this and presented ideas of persecution related to her social exclusion. Her medical history included Graves’ disease discovered at 17, which was treated with Neo-Mercazole without thyroidectomy; the resulting mild hypothyroidism was treated with levothyroxine. She also suffered from astigmatism, myopia, and a keratoconus surgically treated at 18, as well as hyperuricemia. The psychiatric symptomatology suddenly appeared at age 22 without specific trigger event. From age 23, soliloquies and verbigerations appeared, in particular repetitions of sentences in a whispered voice referring to past events (e.g., an argument between neighbors, a potential previous aggression). The family described staring, a social withdrawal, an additional loss of autonomy, as well as sleep disorders. The already present throat clearing worsened and obsessive-compulsive disorders (OCDs) (hand and face washing rituals and drinking rituals) increased. Motor tics (ocular, digital) and verbal tics evocative of Gilles de la Tourette’s syndrome appeared as well as cries, first occasionally, and then progressively every day and at night. At 24, following a consultation with a psychiatrist, treatment with valproic acid was introduced but had no effect on the symptoms. The MRI and EEG were normal. Upon referral to our center, the biological workup revealed highly positive anti- thyroperoxidase (TPO) (>13,000 U/mL), anti-thyroglobulin and anti-thyroid receptor antibodies, leading to a suspicion of steroid-responsive encephalopathy associated with autoimmune thyroiditis (SREAT). She was hospitalized in internal medicine for intravenous corticosteroid boluses over 3 days, followed by an oral corticosteroid therapy at 60 mg/day for 1 month before tapering. Psychomotor function improved with a clear recovery in autonomy, associated with a decrease in anti TPO levels. However the patient was more agitated leading to the prescription of symptomatics low dose antipsychotics. During our last consultation, the parents described their daughter as more lively, having resumed certain games and routines of interaction with them, and considered her condition to be close to that prior to the regression. The corticosteroid therapy is currently at 30 mg/day and 13-fold the anti-TPO antibody.

### Case 3

A 24-year-old man with T21 was referred to our center for anxiety and psychomotor regression that started 4 years ago. His medical history was notable for Hirschsprung’s disease, which required digestive surgery 3 days after birth due to an occlusive syndrome and regular hospitalizations until the age of seven. He also underwent a tonsillectomy in childhood, had thyroiditis with calcifications and obstructive sleep apnea syndrome. There was a language and motor delay (he walked at 24 months). The patient attended ordinary kindergarten, then followed an adapted schooling, before integrating a care home. Until his twenties, the patient was fairly autonomous, able to take public transport alone, and despite his speech difficulties, had no comprehension difficulties. At 21, without any particular event being reported, he progressively developed a loss of spatial orientation, difficulties in his relationships with his peers, and an important susceptibility. He complained of low mood and presented a social withdrawal, a loss of autonomy in public transport due to panic attacks, and subthreshold persecutory delusions. He therefore required permanent help in his journeys. The parents also reported secondary enuresis. In this context, he was diagnosed with major depressive disorder and underwent psychotherapy sessions associated with fluoxetine which initially improved his mood. At 22 years, his relatives described a further loss of autonomy and apathy, the appearance of memory problems, soliloquies, emotional lability, and motor stereotypies. The introduction of sertraline led to a transient behavioral improvement (3 months) but without a return to baseline. Polysomnography found a very disturbed sleep, with apneas and irregular sleep cycles. Continuous positive airway pressure led to improvement in asthenia and memory problems, but he was still no longer autonomous for daily living activities such as washing and dressing. Upon first consultation at the CRMR, the interview was marked by apathy and akinetic mutism. There was no cerebellar syndrome, motor stereotypies, obvious sadness, or psychotic elements. The parents did not report any loss of appetite. On the other hand, the clinical picture was dominated by catatonic elements with maintenance of attitudes, staring, grimacing, negativism, slight grasping, and some extrapyramidal symptoms (stiffness, loss of arm swing). Brain MRI and EEG were normal. The biological workup, done in search of autoimmune and inflammatory syndromes, found an elevated anti-thyroglobulin antibody level (193 IU/ml- three times above the norm). In this context, we progressively introduced lorazepam up to 4 mg/day, which allowed transient improvement of slowness and distractibility. However, because lorazepam was not well tolerated, leading to fatigue, it required an adjustment of the dosage to 2 mg/day then 1 mg/day. The patient remained very slow and mute with graphic perseverations. After unsuccessful lorazepam treatment, we introduced NSAIDs (Naproxen 550 mg x2/d). This strategy improved his speech and writing abilities, as well as his motricity, but only when orders were given sequentially. He remained frozen when he had to self-motivate for actions.

### Case 4

A 22-year-old man with DS was referred to our center for a behavioral regression that started 6 years ago. He presented a history of congenital heart disease with a normal control ultrasound at age 10, hypothyroidism treated with levothyroxine, keratoconus treated with cross-linking at 17, a cataract treated with surgery, and obstructive sleep apnea syndrome treated with continuous positive airway pressure. His mother and sister both had auto-immune thyroiditis. He followed psychomotricity sessions during childhood and orthoptic reeducation for a convergent strabismus. He pronounced his first words at 1 year, started speech therapy at two and a half years, and had a slight stutter. He walked at 36 months. His parents described a child who was always very calm, with difficulty expressing his emotions and pain. He helped with shopping and household chores, was autonomous for daily life gestures (toilet, dressing), and was well oriented in time and space. The patient continued his schooling with individual support in a regular environment during kindergarten and primary school, then in an adapted secondary school. Since childhood, he was very ritualized, presented with motor stereotypies and difficulties in social interactions, especially to maintain the gaze, which suggested autism spectrum disorder. An OCD of alignment, tidying and washing was also reported. Soliloquies were described, but without delusions or verbigerations. At age 16, those close to him reported a behavioral regression with psychomotor slowing, passivity and apragmatism. He could remain still in his bed for hours. Soliloquies and stereotypies increased, echolalia, grimaces, and stiffness appeared. The stammering became more pronounced and hindered communication. No particular event seemed to have occurred that could explain this clinical picture. At age 19, his writing was disrupted. The patient repeatedly went over his writing in a stereotyped way until it became illegible. He also had throat clearing tics, potomania, and sleep disorders with delayed sleep onset, bruxism, and nightly awakenings to eat or drink. In view of a suspicion of polyuro-polydipsia syndrome, a pituitary MRI was performed but came back normal. No agitation, auto-, hetero- aggressive behavior, or delusions were observed. The patient was cooperative during his interactions with his family but his psychomotor slowing was very pronounced. Sometimes maladaptive reactions occurred, such as undirected screams and vocalizations. We first received the patient when he was 21. Clinical examination revealed poor contact with gaze avoidance. He often stared at the ground, with no spontaneous speech, significant latency in answering questions, throat clearing before speaking, and a major stammering that impeded speech intelligibility. There was a global psychomotor slowdown and catatonic symptoms with a frozen attitude, stiffness of the upper limbs and maintenance of a sitting posture with the head and chest bent forward. The Bush-Francis scale score was at 23/69. Brain MRI, EEG, and standard biological workup were normal. Treatment with lorazepam 1 mg led to slight improvement of language and writing. Given the combination of autistic, anxious, and catatonic symptoms, treatment with fluoxetine 20 mg/day was started and then increased to 30 mg/day. In the following months, symptom improvement was noticed with a richer and more fluid speech, and more adapted answers to questions. The patient was more involved in the exchange and gained autonomy. Six months later, we switched fluoxetine 30 mg/day to sertraline 50 mg/day because of hypersudation, but sertraline was less effective. The delayed sleep onset was treated with extended-release melatonin. An autoimmune biological workup showed anti-thyroglobulin and anti-TSH receptor antibodies slightly above the norm. A trial treatment with naproxen showed no efficiency. Oral corticosteroid therapy led to significant but transient improvement in speech difficulties, psychomotor slowing and social anxiety. At our last consultation, the patient partially recovered but still presented slowness and perseveration in oral and written language.

## Discussion

We presented four clinical vignettes illustrating that the symptoms presented by patients with DSRD were multiple and non-specific. All cases exhibited altered mental status, cognitive decline (especially apathy), developmental regression with loss of previously acquired milestones, circadian rhythm disruption, and language deficits. We did not find focal neurological deficits nor seizures but these symptoms may have led to orientation toward a neurologist rather than our psychiatric outpatient unit. Indeed, each patient reported psychiatric symptoms ranging from anxious to psychotic phenomena, although they did not meet full criteria for a psychiatric disorder (subthreshold level of symptoms, fluctuating presentations). In all cases, the response to psychotropic drugs was generally poor, with side-effects, suggesting that the etiology of DSRD should be identified in order to implement specific treatments.

While it is not an etiological diagnosis, catatonia seems to be a syndrome that is often associated with the regression of DS patients (Ghaziuddin et al., 2015). Thus, it could be a relatively common but insufficiently recognized cause of reversible decline in adolescents with DS. Moreover, some hypotheses postulate that DS may be a risk factor for catatonia, independent of DSRD and in addition to those already known such as ASD, mental retardation, and schizophrenia (Ghaziuddin et al., 2015; Miles et al., 2019). Indeed, DSRD could meet the diagnostic criteria for unspecified catatonia not otherwise specified (NOS) according to the DSM-5 criteria (American Psychiatric Association, 2013). Conversely, in our clinical experience, it seems that a diagnosis of catatonia NOS in a patient with DS is in favor of diagnosis of DSRD.

All patients presented with positive autoimmune work-ups, three with autoantibodies directed against the thyroid and one with hypergammaglobulinemia. They responded, at least partially or transiently, to corticosteroid and anti-inflammatory treatments. The hypothesis of an autoimmune or inflammatory etiology in some DSRD therefore seems plausible. Two previously described entities may shed light on this clinical picture. First, PANS has clinical similarities with DSRD as it includes the sudden onset of OCD or restrictive eating disorder, associated with other signs such as abnormal movements, behavioral regression, mood disorder, or anxiety disorder. The etiology of PANS is believed to be an immune response to viral or bacterial infections resulting in the production of antibodies directed to brain structures, which may be responsible for the neuropsychiatric symptomatology. Secondly, SREAT is another rare cause of cognitive decline that may also manifest as catatonia associated with elevated levels of antithyroid antibodies, and may thus have a similar presentation to DSRD. It differs, however, in that convulsions, hallucinations, and ataxia are quite common but not mandatory. It is likely that therapeutic intervention, with anti-inflammatory or corticosteroids, is necessary from the very first signs, in order to prevent the development of the chronic phase of the disorder and allow an optimal recovery.

In line with an inflammatory or autoimmune hypothesis, neuroimaging studies using magnetic resonance spectroscopy based on a glial biomarker demonstrated a higher inflammatory glial component in DS carriers than in control individuals (Hithersay et al., 2017). Many genes involved in microglial activation are also localized on chromosome 21 and thus overexpressed in trisomy (Fortea et al., 2021). Similarly, four of the six interferon receptors are located on chromosome 21, which confers a predisposition in patients with DS to chronic inflammatory reactions and the occurrence of autoimmune pathologies. Recent research revealed the presence of autoantibodies, such as antinuclear antibodies, anti-striated muscle antibodies, anti-TPO antibodies and anti-tissue transglutaminase antibodies, at high levels in some individuals with DSRD (Cardinale et al., 2019; Hart et al., 2021). DS is generally associated with elevated serum levels of proinflammatory cytokines and increased consumption of complement proteins. Autoimmune diseases and in particular Hashimoto’s thyroiditis are common in DS (about 60% of cases) (Miles et al., 2019). In patients with DS, the prevalence of thyroid abnormalities, and in particular hypothyroidism and Hashimoto’s thyroiditis, is 50%, a rate that increases with age (Bull, 2020). Interestingly, patients with DSRD were seropositive for anti-TPO antibodies at a significantly higher rate (91%) than patients of the same age with DS without regression (23%), which may suggest an autoimmune etiology such as SREAT (Worley et al., 2015; Cardinale et al., 2019). Moreover, it is possible that patients with DS may have brains more vulnerable to auto-antibodies compared to the general population, explaining why, in case 4, the patient had more severe consequences than his mother and sister who also exhibit auto-immunity.

These results suggest the involvement of an immune-mediated process in a subgroup of DSRD. Previously reported therapeutic strategies using intravenous immunoglobulins in particular showed significant efficacy in reducing the symptoms of DSRD and support this etiological hypothesis (Cardinale et al., 2019; Hart et al., 2021; Santoro et al., 2022a). Given the clinical response, their use has been proposed in cases of severe and rapid regression, even in the absence of documented autoimmunity (Hart et al., 2021).

There are several limitations that need to be acknowledged. This study is a cases series with a limited number of patients, it is retrospective, and unblinded. Due to this retrospective aspect, we did not perform any specific neuropsychological evaluation, which would allow a better characterization of the treatment effect on cognition. Therefore, a prospective longitudinal approach with quantifiable and standardized outcome measures will be necessary to confirm our tentative observations.

Overall, by highlighting four cases of DSRD, we hope to extend the panel of medical interventions to less invasive strategies. Shortening the delay before adequate treatment might be associated with a better response to NSAIDs or corticosteroids, decreasing the need for more aggressive treatments. Thus, DSRD should be detected early to improve the longitudinal outcome of DS. Awareness of this possible evolution in DS patients, as well as future identification of triggering factors (e.g., stressful events, onset of an auto-immune disorder, infection…) could shorten the use of appropriate therapies.

## Data availability statement

The original contributions presented in this study are included in the article/supplementary material, further inquiries can be directed to the corresponding author.

## Ethics statement

Ethical review and approval was not required for the study on human participants in accordance with the local legislation and institutional requirements. The patients/participants provided their written informed consent to participate in this study. Written informed consent was obtained from the individual(s) for the publication of any potentially identifiable images or data included in this article.

## Author contributions

SB, PE, and BC contributed to the study design. SB contributed to the data gathering. SB, AI, CM, MC, CL, AL, CD-A, CC, AD, AC, M-OK, PE, and BC contributed to the data analysis and interpretation. SB, AI, PE, and BC contributed to the manuscript writing. All authors contributed to the article and approved the submitted version.
